# Push enteroscopy and colonoscopy in melena patients with negative esophagogastroduodenoscopy: Prospective multicenter study

**DOI:** 10.1055/a-2676-3957

**Published:** 2025-08-26

**Authors:** Kotchakon Maipang, Julajak Limsrivilai, Chenchira Thongdee, Arunchai Chang, Kamonthip Sukonrut, Onuma Sattayalertyanyong, Manus Rugivarodom, Uayporn Kaosombatwattana, Nonthalee Pausawasdi, Phunchai Charatcharoenwitthaya, Supot Pongprasobchai

**Affiliations:** 1 65106 Division of Gastroenterology, Department of Medicine, Faculty of Medicine Siriraj Hospital, Mahidol University, Bangkok, Thailand; 2 65106 Siriraj GI Endoscopy Center, Siriraj Hospital, Bangkok, Thailand; 3 65106 Unit of Gastroenterology, Medicine Division, Medical Department, Golden Jubilee Medical Center, Mahidol University Faculty of Medicine Siriraj Hospital, Bangkok, Thailand; 4 37700 Division of Gastroenterology, Department of Internal Medicine, Hatyai Hospital, Songkhla, Thailand; 5 170147 Medicine, Ratchaburi Hospital, Ratchaburi, Thailand

**Keywords:** Endoscopy Small Bowel, Capsule endoscopy, Small intestinal bleeding

## Abstract

**Background and study aims:**

Proper evaluation of patients with melena, no hematemesis, and nondiagnostic esophagogastroduodenoscopy (EGD) is poorly defined. Guidelines recommend colonoscopy, but the additional diagnostic yield is low. Owing to the high likelihood of proximal small bowel bleeding, push enteroscopy (PE) may be beneficial.

**Patients and methods:**

We conducted a prospective, multicenter cohort study from four referral centers. Consecutive patients with melena, no hematemesis, and negative EGD results underwent PE followed by colonoscopy. For patients with culprit lesions found on PE and who were at risk of undergoing colonoscopy, colonoscopy was not performed and results were presumed to be negative. Diagnostic yields of both investigations were compared.

**Results:**

Of 221 eligible patients who underwent EGD, 77 (34.8%) with nondiagnostic results were included in the analyses. Mean age of participants was 67.8 years and 51.9% were men. Culprit lesions were identified on PE in 27 of 77 patients (35.0%). Colonoscopy was performed in 59 patients and the source of bleeding was found in 10 patients (12.9%). Diagnostic yield of PE was significantly greater than that of colonoscopy ( *P* = 0.005). Combining PE and colonoscopy increased diagnostic yield to 48%, which was significantly greater than the yields of PE ( *P* = 0.002) or colonoscopy ( *P* < 0.0001) alone.

**Conclusions:**

PE is beneficial for patients with melena and nondiagnostic EGD. It should be considered before or in combination with colonoscopy for these patients.

## Introduction

Melena is generally considered to result from bleeding in the upper gastrointestinal tract until it is proven otherwise 1 . Consequently, esophagogastroduodenoscopy (EGD) is the preferred procedure for evaluating patients who present with melena. However, EGD fails to identify the bleeding source in approximately one-quarter of patients 2 3 . When EGD is nondiagnostic, evaluation of the mid to lower gastrointestinal bleeding should be considered.

Guidelines recommend colonoscopy before small bowel evaluation in patients with melena and negative EGD results 4 5 . These recommendations are based on studies reporting high detection rates of bleeding sources—23% to 35%—on colonoscopy 6 7 8 . However, these studies are retrospective and have small sample sizes. In contrast, a recent large retrospective study involving 1743 patients with melena and negative EGD findings revealed that colonoscopy identified bleeding sources in only 4.7% of cases 3 .

Moreover, after both EGD and colonoscopy, the cause of overt gastrointestinal bleeding remains undetermined in 4% to 15% of cases. These patients are considered to have potential small bowel bleeding 4 . Guidelines recommend video capsule endoscopy (VCE) for these patients because of its high diagnostic yield and noninvasiveness 4 5 . However, VCE has limitations, including missed proximal small bowel lesions due to rapid transit and inability to perform therapeutic interventions.

Push enteroscopy is another valuable tool for patients with obscure gastrointestinal bleeding. It is a straightforward endoscopic technique that most gastroenterologists can perform without special instruments. In a single retrospective study, the diagnostic yield of push enteroscopy in patients with melena was reported to be 40% 9 . Therefore, selecting push enteroscopy for patients with melena and negative EGD findings may be reasonable. This approach could help avoid unnecessary colonoscopy and VCE, potentially reducing treatment costs. We conducted a prospective study to evaluate diagnostic yields of push enteroscopy and colonoscopy in patients presenting with melena without hematemesis and with negative EGD results.

## Patients and methods

### Study design and setting

We conducted a prospective, multicenter cohort study from July 2019 to October 2022 at four referral centers in Thailand. They were the Faculty of Medicine Siriraj Hospital, Mahidol University, Bangkok; Golden Jubilee Medical Center, Mahidol University, Bangkok; Hatyai Hospital, Songkhla; and Ratchaburi Hospital, Ratchaburi.

### Patient selection

Consecutive patients presenting with melena (black, tarry stools) without hematemesis were invited to participate. Inclusion criteria were age 18 years or older and underwent EGD without bleeding source identified. Exclusion criteria were the following: 1) unstable hemodynamics defined by blood pressure lower than 90/60 mm Hg despite receiving an inotropic agent prior to push enteroscopy; 2) respiratory compromise defined by desaturation requiring high flow oxygen or mechanical ventilator; 3) uncorrectable bleeding disorders; and 4) pregnancy.

### Data collection and procedures

Baseline demographic data, comorbidities, and bleeding characteristics and severity were recorded. Patients underwent push enteroscopy followed by colonoscopy. Time from presentation to each endoscopic procedure and the findings were documented. Culprit lesions—identified as the most likely cause of bleeding—were detailed and included ulcers, angiodysplasia, Dieulafoy’s lesions, tumors, diverticula, and other abnormalities.

Colonoscopy was not performed for patients whose push enteroscopy detected culprit lesions and were deemed at moderate to high risk for developing colonoscopic complications, including significant anesthetic risk or multiple comorbidities. In this group, the colonoscopic results were presumed to be negative, and a 3-month follow-up without rebleeding was applied to confirm absence of additional bleeding sources beyond those identified by push enteroscopy. For patients with negative results from both push enteroscopy and colonoscopy, further investigations were conducted at the discretion of the treating physicians. Diagnostic and therapeutic yields of push enteroscopy and colonoscopy were compared.

### Push enteroscopy procedure

Push enteroscopy was performed using PCF-Q180AL or PCF-HQ190L/I pediatric colonoscopes (Olympus Medical Systems Corporation, Tokyo, Japan). Patients fasted for 6 to 8 hours before the procedure to optimize gastrointestinal visualization. Sedation with intravenous propofol ensured patient comfort and safety. If necessary, hyoscine butyl-bromide was administered to reduce small bowel motility. The endoscope was inserted orally and advanced through the esophagus, stomach, and duodenum to reach the proximal small intestine. Minimal insufflation was used to reduce discomfort and maintain clear mucosal visualization. Air was used for insufflation during the procedure. CO _2_ was not used to ensure uniformity of practice and consistency of care across all participating centers. Careful inspection for abnormalities was conducted during scope withdrawal. Patients were monitored during recovery and provided with clear recuperation instructions.

### Statistical analysis

Descriptive statistics summarized patient characteristics. Continuous variables are expressed as means and standard deviations or as medians and interquartile ranges (IQRs); categorical variables are presented as counts and percentages. Standard two-group comparison methods were used, including an independent t-test (for normally distributed data) or Wilcoxon rank-sum test (for non-normally distributed data) for continuous data, and a chi-square test or Fisher exact test (depending on sample size) for categorical data. The McNemar test was used to assess differences in diagnostic yield among push enteroscopy, colonoscopy, and their combination. Statistical analyses were performed via SAS OnDemand for Academics (SAS Institute, Cary, North Carolina, United States). A two-tailed *P* < 0.05 was considered statistically significant.

### Ethical considerations

All patients provided written informed consent after receiving detailed information about the diagnostic procedures. The study was approved by the local institutional human research review committee (approval number Si-484/2019) and conformed to good clinical practices. The research was registered with ClinicalTrials.gov (NCT06574542).

## Results

### Patient characteristics

A total of 221 patients presented with melena without hematemesis and underwent diagnostic EGD, which identified bleeding sources in 141 patients (63.8%). Three patients were excluded owing to respiratory compromise. The remaining 77 patients (34.8%) with nondiagnostic EGD results were included in our analyses. The mean age of these patients was 67.8 years and 51.9% were men ( Table 1 ).

**Table:** **Table 1** Baseline characteristics of the study population.

Parameters	N = 77
Demographic data	
Age, mean ± SD	67.8 ± 14.8
Sex, male (%)	40 (51.9%)
Comorbidities, n (%)	
Hypertension	50 64.9%)
Diabetes mellitus	26 (33.8%)
AF or valvular heart disease	20 (26.0%)
Coronary artery disease	15 (19.5%)
Chronic kidney disease	26 (33.8%)
Dialysis	10 (13.0%)
Cirrhosis	3 (3.9%)
Medications	
NSAIDs	7 (9.1%)
Antiplatelets	21 (27.3%)
Anticoagulants	19 (24.7%)
Warfarin overdose	5 (6.5%)
Clinical presentations	
Hypotension	9 (11.7%)
Syncope	15 (19.5%)
Tachycardia	23 (30.3%)
Previous bleeding	
Number of previous bleeding episodes	18 (23.4%)
PRC transfusions	
Number of PRC transfusions	2.8 ± 2.0
Laboratory investigations	
Hemoglobin, g/dL	6.5 ± 1.9
INR	1.6 ± 1.4
BUN, mg/dL	40.5 ± 31.6
Cr, mg/dL, median (IQR)	1.1 (0.9 - 2.1)
BUN/Cr ratio, mean ± SD	26.5 ± 20.0
AF, atrial fibrillation; BUN, blood urea nitrogen; Cr, creatinine; INR, international normalized ratio; IQR, interquartile range; NSAID, nonsteroidal anti-inflammatory drug; PRC, packed red cell; SD, standard deviation.	

The most common comorbidities were hypertension (64.9%), diabetes mellitus (33.8%), chronic kidney disease (33.8%), atrial fibrillation or valvular heart disease (26.0%), and coronary artery disease (19.5%). In addition, 9.1% of patients had a history of nonsteroidal anti-inflammatory drug use, 27.3% had used antiplatelets, 24.7% had used anticoagulants, and 6.5% had experienced warfarin overdose. Almost one-quarter (24.7%) had a history of previous bleeding. No patients with surgically altered upper gastrointestinal anatomy were enrolled.

### Endoscopic procedures and timing

All 77 patients underwent push enteroscopy, and 59 patients proceeded to colonoscopy. Median times from hospital presentation to EGD, push enteroscopy, and colonoscopy were 3.0 days (interquartile range [IQR] 1.0–5.0), 3.0 days (IQR 2.0–6.0), and 5.0 (IQR 3.0–7.0), respectively.

Mean procedure time was 27.9 ± 13.9 minutes for push enteroscopy and 31.4 ± 28.7 minutes for colonoscopy. No serious complications such as bleeding or perforation occurred during either push enteroscopy or colonoscopy.

### Diagnostic yield of push enteroscopy and colonoscopy

**Fig. 1:**
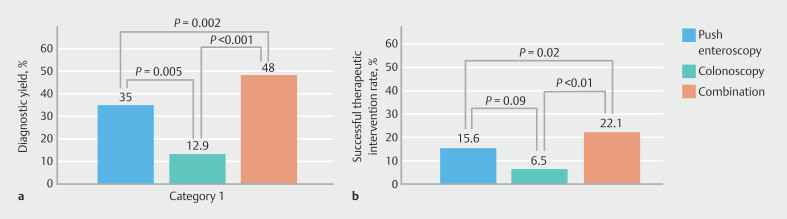
**a** Diagnostic yield and **b** therapeutic intervention success rate for push enteroscopy and colonoscopy in melena patients with negative esophagogastroduodenoscopy results.

**Fig. 2:**
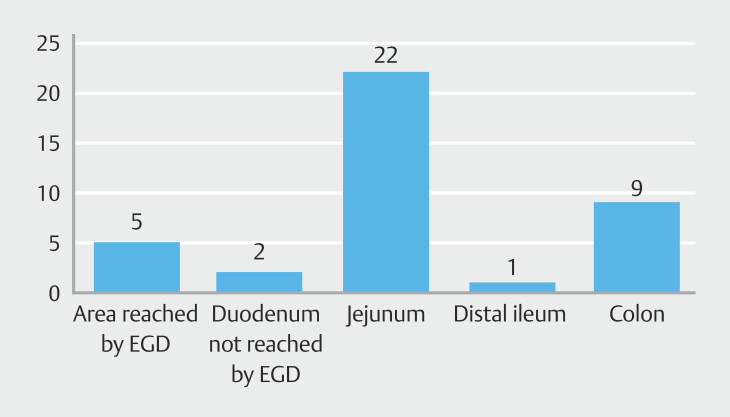
Locations of lesions identified by push enteroscopy in melena patients with negative esophagogastroduodenoscopy results. EGD, esophagogastroduodenoscopy.

As shown in Fig. 1 **a** and Table 2 , 27 of 77 patients (35.0%) had a culprit lesion identified during push enteroscopy, including ulcers (25.9%), angiodysplasia (29.6%), Dieulafoy’s lesion (11.1%), tumor (14.8%), polyps (3.7%), bleeding diverticulum (3.7%), and other conditions (11.1%). Locations of lesions were at the duodenum reached by EGD in five patients, the duodenum not reached by EGD in two patients, and the jejunum in 22 patients ( Fig. 2 ). Five patients (18.5%) had lesions missed during previous EGD. Lesions comprised duodenal ulcers, duodenal angiodysplasia, and hemosuccus pancreaticus. Colonoscopy was performed in 59 cases (76.6%) and bleeding sources were identified in 10 patients (12.9%). Culprit lesions found during colonoscopy were ulcers (3 patients), Dieulafoy’s lesions (3 patients), cancer (1 patient), polyp (1 patient), diverticulum (1 patient), and colitis (1 patient).

**Table:** **Table 2** Types and locations of lesions detected by push enteroscopy and colonoscopy.

**Characteristics**	**Total (N = 37)**	**Push enteroscopy (N = 27)**	**Colonoscopy (N = 10)**
Type of lesions			
Ulcers	10 (27.0%)	7 (25.9%)	3 (30.0%)
Angiodysplasia	8 (21.6%)	8 (29.6%)	0 (0.0%)
Dieulafoy’s lesion	6 (16.2%)	3 (11.1%)	3 (30.0%)
Tumor	5 (13.5%)	4 (14.8%)	1 (10.0%)
Polyp	2 (5.4%)	1 (3.7%)	1 (10.0%)
Diverticulum	2 (5.4%)	1 (3.7%)	1 (10.0%)
Other	4 (10.8%)	3 (11.1%)	1 (10.0%)

Diagnostic yield of push enteroscopy was significantly greater than that of colonoscopy ( *P* = 0.005). Combining push enteroscopy and colonoscopy increased the overall diagnostic yield to 48% (37 patients), which was significantly greater than the yield of push enteroscopy alone ( *P* = 0.002) or colonoscopy alone ( *P* < 0.0001).

### Univariate analysis of predictive factors for positive findings on push enteroscopy

Univariate analysis was performed to identify predictive factors associated with positive findings on push enteroscopy ( Table 3 ). Variables analyzed included age, sex, underlying comorbidities, medication use, and blood urea nitrogen/creatinine ratio. No significant associations were found between these factors and positive findings on push enteroscopy.

**Table:** **Table 3** Univariate analysis of predictors for positive findings in push enteroscopy.

**Parameters**	**Odds ratio (95% CI)**
Age	1.00 (0.97–1.04)
Sex, male	1.25 (0.49–3.20)
CKD	1.25 (0.47–3.33)
AF or valvular heart disease	1.33 (0.47–3.81)
NSAIDs	2.72 (0.56–13.19)
Aspirin	0.73 (0.24–2.20)
Antiplatelets	0.66 (0.22–1.98)
Warfarin	0.91 (0.27–2.99)
Time to push enteroscopy	1.06 (0.94–1.19)
Hypotension	0.91 (0.21–3.99)
Hemoglobin	1.02 (0.80–1.31)
BUN/Cr	1.00 (0.98–1.03)
AF, atrial fibrillation; BUN, blood urea nitrogen; CI, confidence interval; CKD, chronic kidney disease; Cr, creatinine; NSAID, nonsteroidal anti-inflammatory drug.	

### Therapeutic interventions and outcomes

Therapeutic interventions were conducted during push enteroscopy in 13 patients (16.9%) and during colonoscopy in five patients (6.5%). Successful interventions were achieved in 12 patients (15.6%) during push enteroscopy and in all five patients (6.5%) during colonoscopy. As shown in Fig. 1 **b** , the successful therapeutic intervention rate was greater for push enteroscopy than for colonoscopy, although this difference was not statistically significant ( *P* = 0.089). Compared with either method alone, combined use of push enteroscopy and colonoscopy significantly increased the successful therapeutic intervention rate.

The 30-day rebleeding rate was 3.7% for patients who received therapeutic intervention via push enteroscopy and 0% for those treated via colonoscopy.

### Further investigations and definitive diagnoses

Further investigations were performed for patients whose bleeding sources were not identified or managed. These were VCE in 10 patients, computed tomography in six patients, and balloon-assisted enteroscopy (BAE) in four patients.

A definitive source of bleeding was ultimately identified in 53 patients (68.8%) through combined use of initial procedures and further investigations.

## Discussion

Melena results from degradation of hemoglobin and implies bleeding from the upper gastrointestinal tract. Therefore, EGD should be the first-line investigation in patients presenting with melena 2 3 . Our findings support this recommendation: In 64% of patients with melena, bleeding sources were revealed via EGD. A previous study reported that approximately three-quarters of these patients had lesions detected on EGD 10 .

After negative EGD, guidelines recommend performing colonoscopy in patients presenting with melena before proceeding to small bowel evaluation 4 5 . Our study is the first prospective analysis to evaluate diagnostic yield of colonoscopy after negative EGD in patients with melena. We found that the diagnostic yield was 14%, which is lower than the previous retrospective reports of 23% to 35% 6 7 8 . Although the diagnostic yields were not high, 5% to 10% of the lesions detected in previous studies and our study were colon cancers 10 11 . Furthermore, colonoscopy can be used to perform therapeutic interventions. In our study, therapeutic interventions were performed on five of 10 lesions detected during colonoscopy and all the treatments were successful. Given the benefits of cancer detection, therapeutic capability, and accessibility, we believe that colonoscopy should still be considered in patients with melena despite its fair diagnostic yield. However, more investigations are likely to be required.

VCE is a noninvasive modality that standard guidelines recommend as the first-line investigation after negative EGD and colonoscopy; the condition is defined as potential small bowel bleeding 4 5 . VCE has a high diagnostic yield of 55% to 65% for overall potential small bowel bleeding. However, data regarding its yield specifically for patients with melena, not hematochezia, are limited. Only one study by Mussetto et al reported the VCE yield in patients with melena and negative EGD results using a panenteric capsule in 12 patients. Bleeding sources were found in 10 patients (83.3%). Besides limited data on VCE in this specific setting, VCE has several limitations. First, it can miss proximal small bowel lesions due to rapid transit; the major papilla in the second part of the duodenum is visualized in only 10.4% to 43.6% of patients 12 13 . Second, VCE cannot perform therapeutic interventions. Some lesions, such as Dieulafoy’s lesion and angiodysplasia, may not actively bleed and cannot be identified during subsequent direct enteroscopy. Early direct enteroscopy could be beneficial for these vascular lesions. Interestingly, small bowel angiodysplasia has been reported to be predominantly located in the proximal small bowel. Specifically, studies have reported that 67% of small bowel angiodysplasia lesions are located in the duodenum or ligament of Treitz 14 . Therefore, an antegrade approach for small bowel enteroscopy is a reasonable option. This technique can overcome the limitations of VCE in missing proximal small bowel lesions and the inability to provide therapeutic intervention for vascular lesions.

Given the substantial possibility of bleeding in the proximal small bowel in the setting of melena, push enteroscopy should play a role. Limited evidence has demonstrated the performance of push enteroscopy in patients with melena. Most previous trials included all potential cases of small bowel bleeding—either melena or hematochezia, reporting a diagnostic yield of 19% to 35% 15 16 17 18 . Only a study by Lepère et al 9 reported the diagnostic yield specifically in patients with melena. In their retrospective study, the overall diagnostic yield of push enteroscopy for all potential small bowel bleeding cases was 34%. The yield increased to 40% in patients presenting with melena and decreased to 17% in patients presenting with hematochezia. Our study is the first prospective analysis to evaluate diagnostic yield of push enteroscopy in patients presenting with melena and negative EGD results. We demonstrated that diagnostic yield of push enteroscopy was 35% in these patients.

Another benefit of push enteroscopy is its ability to perform therapeutic interventions. In our study, 13 of 27 patients whose bleeding sources were detected received interventions and 12 of these interventions were successful. Furthermore, push enteroscopy is safe; no serious complications were observed in our study. Push enteroscopy is also a simple endoscopic technique that does not require special instruments and most endoscopists can perform it. Thus, push enteroscopy should be considered in patients with melena and negative EGD findings. The American College of Gastroenterology clinical guidelines also recommend push enteroscopy as an option in patients with gastrointestinal bleeding who have negative EGD and colonoscopy results before VCE 4 . Another advantage of using push enteroscopy as the initial investigation is that it may reduce need for bowel preparation, which is typically required for colonoscopy. This is particularly relevant for hospitalized patients, in whom bowel prep is known to be more frequently suboptimal compared with outpatient settings. This can contribute to lower diagnostic yield of colonoscopy in this population 19 20 .

The drawback of push enteroscopy is its limited reach: It can access the small bowel only approximately 0.6 meters distal to the duodenojejunal junction. Melena can originate from bleeding at more distal parts 21 . Therefore, identifying predictors for proximal small bowel bleeding is important in selecting suitable patients for push enteroscopy. A study by Lepère et al revealed that chronic renal failure was a predictor associated with positive findings on push enteroscopy 9 . Unfortunately, our univariate analysis did not identify any significant predictive factors associated with positive findings on push enteroscopy. Variables analyzed included age, sex, underlying comorbidities, medications used, and blood urea nitrogen/creatinine ratio.

BAE has a high diagnostic yield comparable to that of VCE in patients with potential small bowel bleeding 22 23 24 . Compared with push enteroscopy, deeper small bowel insertion leads to a greater diagnostic yield 25 26 . Furthermore, BAE can perform therapeutic interventions. Therefore, BAE may be an appropriate option for investigating patients with melena. However, BAE carries risks of complications, including sedation-related issues, bleeding, perforation, and pancreatitis 27 28 . In addition, BAE is unavailable at many centers and requires highly skilled endoscopists, limiting its widespread use.

Given these considerations, we believe that push enteroscopy should be performed in patients who present with melena and negative EGD results. It could be considered before colonoscopy. However, these two modalities complement each other. Our study revealed that combining push enteroscopy and colonoscopy increased the diagnostic yield to 48%, which was significantly greater than that of either method alone. Furthermore, therapeutic intervention rates were significantly higher for the combination than for each modality. Therefore, if push enteroscopy is negative, colonoscopy should be performed afterward. After negative push enteroscopy and colonoscopy, VCE should be performed, and BAE should be considered according to the VCE findings.

This study has several strengths. It is the first prospective investigation of consecutive patients who presented with melena without hematemesis and negative EGD results. We assessed diagnostic and therapeutic yields of both push enteroscopy and colonoscopy. In addition, therapeutic interventions were performed in the same or nearly the same session, providing timely treatment when necessary.

However, there are several limitations. Colonoscopy was not performed for every patient because of safety concerns. For patients whose culprit lesion was identified during push enteroscopy and who had heightened health risks, colonoscopy was sometimes omitted to avoid unnecessary procedural risks. This omission could have resulted in missing additional lesions. To address this issue, we conducted 3-month follow-up to ensure no recurrent bleeding. We believe that clinically significant lesions would likely have manifested as recurrent bleeding or anemia during this period, enabling timely detection and management. Although there is no universally established guideline for optimal duration of follow-up, randomized controlled trials in patients with lower gastrointestinal bleeding have used a shorter duration of 30-day rebleeding as a secondary outcome 29 30 .

Furthermore, a significant proportion of patients in our study were recruited from large tertiary referral centers, which may introduce referral bias. These institutions often care for patients with more severe comorbidities or refractory gastrointestinal bleeding, which may not reflect the typical case mix encountered in general or community hospital settings. Consequently, diagnostic yields reported in this study may not be fully generalizable to broader clinical populations.

Finally, our findings are intended to complement current diagnostic strategies for patients with suspected small bowel bleeding. Push enteroscopy may serve as a valuable adjunctive tool that enhances diagnostic and therapeutic outcomes, especially in settings where it is available, rather than acting as a replacement for established diagnostic modalities.

## Conclusions

In conclusion, this study confirms the essential role of EGD in evaluating patients who present with melena despite absence of hematemesis. If EGD is negative, push enteroscopy should be considered before, or in combination with, colonoscopy. Future prospective studies with larger sample sizes are warranted to allow for more robust statistical analyses and validation of these findings.
